# Recommendations for procedures related to the evidence chain of custody in forensic anthropology in Brazil

**DOI:** 10.1080/20961790.2022.2076984

**Published:** 2023-02-12

**Authors:** Nicole Prata Damascena, Melina Calmon Silva, Alexandre Raphael Deitos, Rosane Pérez Baldasso, Renata Cristina Grangeiro Ferreira, Cristian Kotinda Júnior, Carlos Eduardo Palhares Machado

**Affiliations:** a Departamento de Polícia Técnica da Bahia, Bahia, Brazil; b Grupo de Pesquisa em Antropologia Forense e Identificação de Pessoas, Brasilia, Brazil; c Instituto Nacional de Criminalística da Polícia Federal, Brasilia, Brazil; d Departamento Médico-Legal do Instituto-Geral de Perícias do Rio Grande do Sul (IGP/RS), Porto Alegre, Brazil; e Instituto de Criminalística da Polícia Civil do Distrito Federal, Brasilia, Brazil.

**Keywords:** Forensic sciences, forensic anthropology, chain of custody, evidence, law, Brazil

## Abstract

Forensic anthropologists perform specialised analysis, mainly involving skeletonised human remains, cadavers in advanced stages of decomposition, disassociated elements from the human body, and human remains in extreme carbonisation. The main objectives of the forensic anthropology expertise are human identification and assisting in determining the cause and manner of death. Estimating the time since death is also a priority for some cases, especially ones involving missing persons. This science works on individual cases, violent deaths, missing persons, mass disasters, suspected violations of human rights, and crimes against humanity. Forensic anthropological evidence is, in general, very sensitive. Thus, it is important to detail aspects relevant to the maintenance of the chain of custody at all phases of the investigation, as well as standardise the actions of the individuals involved. This aims to preserve the evidence integrity and sameness (Sameness: free translation of the Portuguese word “mesmidade”, derived from a Spanish word that does not possess a translation to Portuguese. Sameness of evidence is understood as the guarantee that the evidence under valuation (or under analysis of its probative value) is exactly and integrally the same one which was collected, corresponding, therefore, to “the same” (and not “part of”, “derived from”), safeguarding its value. Brazil Law No. 13.964 of 14 December 2019 establishes and lists 10 phases related to the evidence chain of custody that must be followed. These newly introduced requirements resulted in the need for adaptation of the forensic, investigative, and legal actors involved in an investigation, and in the detailed description of the procedures for the different areas related to criminalistics, including forensic anthropology. The information provided in this article should be interpreted as recommendations, even though their non-compliance may weaken the investigative and forensic analysis processes in whole or in part.

## Introduction

The establishment of the term *chain of custody* is part of a relatively recent process in the history of criminalistics. In the USA, the first concerns with the chain of custody emerged in the mid-1900s. However, there was no understanding regarding its use and obligation. Countless judges dismissed its need in favour of testimonial evidence or positive identification of real evidence historically related to the case [1].

Later, the theme gained more prominence with the book and episode: “The People Against OJ Simpson”, a case involving a famous former American football player, Orenthal James Simpson, accused as the main suspect in the murder of his ex-wife (Nicole Brown) and his friend (Ronald Goldman) in 1994 [2]. In the case in question, several chain of custody procedures were ignored, and the exposed flaws had repercussions worldwide. This emphasised the importance of the subject and manifested a substantial increase in the number of discussions regarding it.

Consequently, numerous and varied chain of custody concepts emerged. The International Organization for Standardization (ISO), through its standard 22095:2020, defined the chain of custody as “*process by which inputs and outputs and associated information are transferred, monitored and controlled as they move through each step in the relevant supply chain*” [3]. In another ISO standard, 21043-1:2018, part 1, which deals with forensic sciences (terms and definitions), the chain of custody is defined as “*chronological record of the handling and storage of an item from its point of collection to its final return or disposal*” [4]. Both ISO concepts are similar to those proposed in the Brazilian Code of Criminal Procedure [5].

The involvement of Brazil in the discussions regarding chain of custody started in the first decade of the 19th century [6, 7]. But the issues that permeate the preservation and reliability of evidence arrived much earlier than the Code of Criminal Procedure in force and the legislations that further changed or added to it. The discussions regarding elements that are now seen as part of evidence chain of custody have been debated and detailed in the forensic investigative realm since the 1940s, when the term criminalistics was first adopted in Brazil. However, what we experience today is an effort to respond to a legal demand (Law No. 13.964/2019) that has made it mandatory to follow best practices that in the past were seen as recommendations in the forensic sphere.

In a way, the concern with “forming the *corpus delicti*” as soon as the fact is known is as old as the period of the Brazilian Empire. In the Code of Criminal Procedure of 1832, it is observed that the legislator was already concerned regarding the safeguarding of the element and its provable characteristics.

Since then, other texts have also addressed the subject, and this is evidently reflected in the current doctrine. One of the forensic texts that summarises the question appears in reference [8], under the title of “Defense, Labeling and Consignment of Collected Material”. The author defines that:

By “defense of the material” we must understand its protection, so that nothing in it is lost, nothing in it is altered, allowing a perfect expert examination.

(Porto, 1969)

The relationship between what the author defined and what we understand today as an evidence chain of custody is clear. More than that, in line with Dias Filho [7], Porto [8] pointed out that “*This defense begins when the ‘scene’ is isolated. Isolation must be carried out by the policeman, in order to preserve the scene as he/she/they found it when he/she/they arrived*”^1^. Porto [8] continued and described the importance of the evidence collection, packaging, and labelling.

The coherence between the forensic recommendations of Porto [8] and the text sanctioned by Law No. 13.964/2019 with 50 years between them is remarkable. Yet, the important point is that the concern with maintaining the evidentiary characteristics of a trace evidence is not new in Brazil, despite having taken at least five decades to be more clearly incorporated into the national criminal procedural system.

The National Secretariat for Public Security (SENASP), bound to the Ministry of Justice and Public Security (MJSP), published Ordinance No. 82 on 16 July 2014, which established the guidelines for the procedures to be observed regarding the evidence chain of custody [9]. The regulation, however, was only mandatory for the National Public Security Force and did not impact the forensic, investigative, or legal procedures in Brazil as a whole.

An important contemporary Brazilian legal scholar on the subject, Prado [10], stated that the evidence chain of custody is a method of preserving the integrity and authenticity of the evidential element. He also said that a violation of the chain of custody implies the impossibility of the valuation of the evidence (provable value analysis), configuring one of the objects of the judgment of admissibility of the means of evidence or the means of obtaining evidence, which is not subject to judgment of evidential weight nor evidential relevance. He concluded that there is no doubt that the chain of custody is a condition for the prosecution of the *corpus delicti* examination.

Notwithstanding Prado’s arguments, part of the legal doctrine understands, however, that the breach of the evidence’s chain of custody should be dimensioned in the valuation stage. The seriousness of the breach and the degree of effective contamination of its reliability should be assessed, so that there is the possibility of its admissibility to a greater or lesser extent [11].

Bill No. 6.341 of 2019 proposed an improvement in forensic and criminal procedural legislation [12], culminating in the enactment of Law No. 13.964 of 24 December 2019 in the so-called *Anti-Crime Package* [13]. Among other changes, the package inserted the chain of custody in Chapter II of Title VII of the Code of Criminal Procedure, renamed as “Examination of the c*orpus delicti*, the chain of custody and forensics in general”. In fact, practically the entire content of the SENASP ordinance was reproduced in the legal amendments implemented in 2019.

When discussing the new Law No. 13.964/2019 that first addressed the phases and their descriptions on the evidence chain of custody in the Brazilian legal system, it may seem that the chain of custody theme is innovative in Brazil. However, as mentioned, the implementation of procedures related to the chain of custody in an avowed way in the law is just another advance in the modernisation of the Brazilian criminal procedural legislation regarding the preservation and reliability of evidence.

The term *chain of custody* was legally defined as:

…the set of all procedures used to maintain and document the chronological history of the trace collected in places or victims of crimes, to track its possession and handling from its recognition to the disposal.

(Article 158-A of Law No. 13.964 of 2019)

The term, in practice, aims to provide credibility and authenticity to the material provided as evidence, with specified characteristics to dispel doubts in its origin and movement. Therefore, currently, the chain of custody is the most critical process in the documentation of evidence and its importance can be evaluated by the quantity and quality of national and international publications and guidelines about its practice.

In this context, the “*Chain of Custody Technical Group Project: discussion, diagnosis, and recommendations after Law No. 13.964/2019*” was developed, instituted through SENASP/MJSP Ordinance No. 282 on 21 May 2021, in the scope of the Working Group on the same topic, established through SENASP Ordinance No. 176 on 29 September 2020 [14]. The objective was to conduct structured and systematised technical discussions with specialists and multidisciplinary groups involved with the identification, security, documentation, collection, packaging, transportation, reception, processing, storing, and disposal of evidence and traces potentially related to criminal offenses.

Composed of nearly 150 professionals from multiple National Public Security institutions organised into 13 Technical Groups (TGs), the project’s team had the challenging mission of discussing procedures and mapping processes in the chain of custody in a national approach and multi-agency manner. It had to consider the operational, tactical, and strategic levels, with the goal of issuing recommendations and producing workflows that adapt the plurality and heterogeneity of National Public Security institutions to the minimally acceptable criteria of chain of custody.

Given the above, this article aims to expose the discussions, diagnoses, and recommendations that encompass the work of the TG entitled Forensic Medicine, Toxicology, Forensic Anthropology, and Legal Odontology (TG MLTAFOL) and the General Coordination and Integration Group (CGI), based on extensive technical references on the subject. Such data and information presented here come from the integrated and multidisciplinary work of the members of the group. This work and the one in which this article is based, is the first to develop recommendations for establishing parameters for the chain of custody at a national level.

Although the authors recognise that the discussions happened in a broader sense encompassing different forensic specialties, our goal here is to stress these recommendations in the practice of forensic anthropology in Brazil. The authors understand that some of the information exposed here can be extended to other forensic-related areas that deal with human remains, but we also know that there is variation across disciplines and their scope. Therefore, because of the depth of the discussions, we advise that any extrapolations outside the scope of forensic anthropology are made with their area-specific adjustments.

## Forensic anthropology and the Medico-Legal Institutes in Brazil

Forensic anthropology analyses in Brazil are normally performed within the Medico-Legal Institutes (IMLs) in conjunction with the Institutes of Criminalistics (IC), inserted as part of the attributions of the official forensic experts. The forensic medicine experts and legal odontology experts are typically the ones responsible for the forensic anthropological examinations. The Federal Law No. 12.030/2009 addresses those who are the official forensic experts of criminal nature, namely: criminal experts, forensic medicine experts, and legal odontology experts [15]. As a rule, there are no specific criteria and training requirements for these experts to work in forensic anthropology, with an overall lack of training and standardisation of procedures in the field in the country coupled with a lack of guidelines regarding the necessary procedures for the chain of custody.

The discussions regarding the chain of custody in forensic anthropology were first addressed in the TG MLTAFOL because these areas are all included in the IMLs in Brazil. The purpose of the established document was to ensure the effectiveness of the chain of custody of traces of Legal Medicine, Legal Odontology, Forensic Anthropology, and Forensic Toxicology interest in the phases of identification, security, documentation, collection, packaging, transportation, reception, processing, storing, and disposal of evidence. It also aimed to standardise the actions of the personnel involved to preserve the inviolability and suitability of the traces, safeguarding their evidential value.

**Figure 1.**
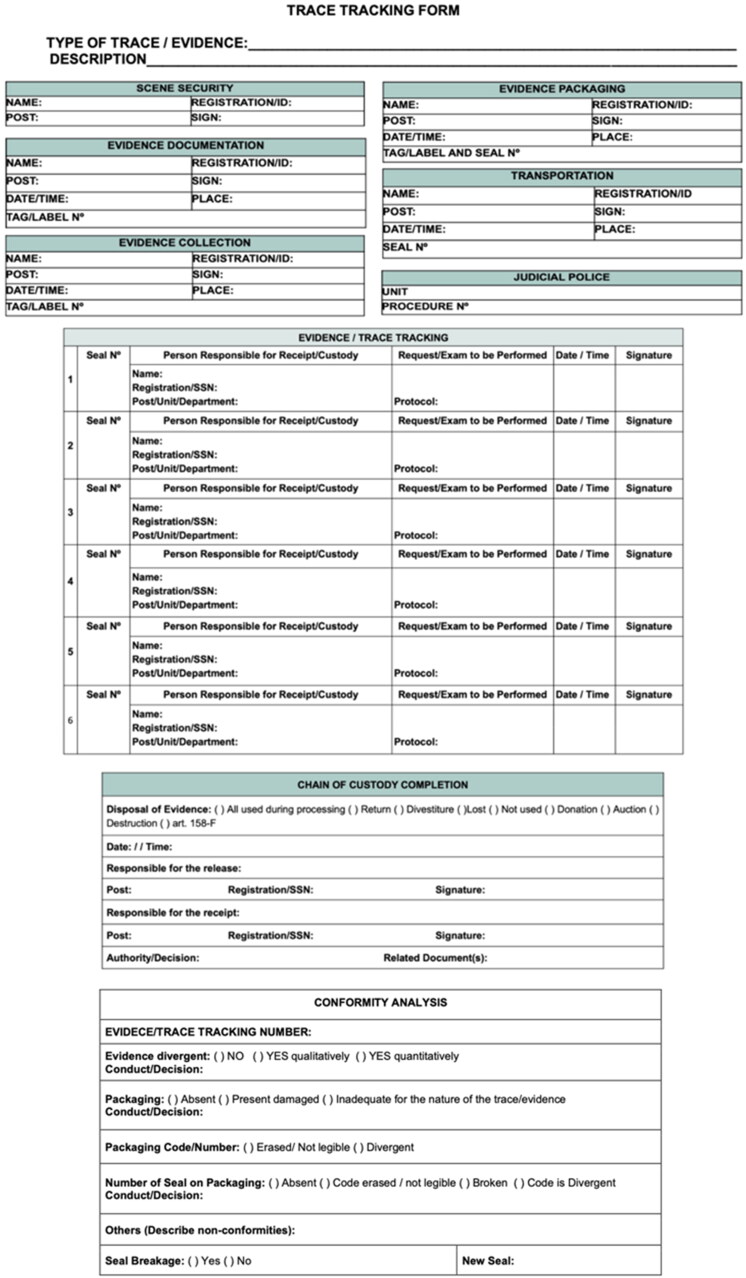
Model of a Trace Tracking Form (TTF). This model is an example and can be changed or adapted. The data and information shown must be available so that there is a registration of the people who were possibly in contact with the evidence. Ideally, the TTF is in digital form in addition to a hard copy (when a bar code is present on the paper copy) or as a stand-alone digital procedure.

In this sense, the Trace Tracking Form (TTF; FAV in Portuguese) (Figure 1) is at the heart of the chain of custody and must: (i) have exclusive numbering; (ii) be completed in physical or digital media; and (iii) monitor the trace throughout its processing until its final destination. The TTF has items corresponding to each stage of trace processing, divided into sub-items that aim to optimise trace tracking in specific phases considered more sensitive. In the TTF, all the steps of monitoring the human remains and/or associated evidence, such as the placement and subsequent breaking of the seal and the new numbers, must also be registered, as well as the identification of those responsible for such alterations. It is important to remember that after each break of the seal, the old one must be packed inside the new trace bag to be sealed.

The examinations and data collection in a crime scene involving human remains are used to reconstruct and interpret the past events that led to the presence of a deceased individual. The information available on the scene, along with the human remains, are then referred for the examination at the IMLs in Brazil. The presence of professionals specialised in forensic anthropology at crime scenes is of great value for acting directly in the survey, mapping, and excavation processes (when needed) and the recovery of physical evidence [16]. However, this is far from becoming a routine in Brazil. In fact, professionals who work with forensic anthropology at IMLs are rarely present at crime scenes. They often receive at their laboratory the evidence collected by other professionals who are not aware of the necessary specificity of evidence that has forensic anthropological significance.

Because the maintenance of the chain of custody goes beyond the mere traceability of a piece of evidence and applying proper methods and techniques by specialised personnel to guarantee the sameness and evidential value of the trace, proper personal protective equipment (PPE) must be always used. Proper PPE depends on the stage of decomposition of the identified, collected, and analysed remains, but should ideally minimally include gloves, cap, surgical mask, apron or protective overalls, and shoe protectors.

One objective of the IMLs is to establish the cause and manner of death and human identification. They should preferably allocate a physical space aiming at the custody and control of evidence that has forensic anthropological significance. These should comprise sections exclusively dedicated to the reception, processing, and storage of human body elements, human remains, dental elements, and bone fragments. The IML Custody Unit^2^ is designed to store and control these types of evidence and ideally consists of three sections under the responsibility of IML employees:

- General Evidence Custody Unit: for receiving, processing, and storing evidence associated with human remains (personal effects, for instance);
- Cold Chamber Custody Unit (Morgue): intended for receiving, processing, and storing cadavers;
- Ossuary Custody Unit: for receiving, processing, and storing skeletonised remains, bone elements, dental elements, and bone fragments.

In the internal structure of the IML focused on forensic anthropology, it is desirable that there is a specific Custody Centre for bones, herein called “Ossuary Custody Centre”. This should consist of a dry and airy room for the storage of urns, boxes, or bags with identified/numbered shelves to enable the efficient classification and searching of evidence, including isolated dental elements and bone fragments. Thus, when the forensic anthropological examination is completed, the bone elements or skeletonised human remains should be sent to the Ossuary Custody Centre for temporary or permanent custody. In the absence of expert and criminal interest, the recommended disposal rite for the release of the remains will be followed (described in more detail later in this article).

## Discussions and recommendations related to the phases of the chain of custody in Brazil

The following discussions were based on the compilation of references mentioned previously in this article. Therefore, as we list the 10 phases that make up the chain of custody processes with their legal definitions, as referenced in Law No. 13.964 of 2019, the applications to forensic anthropology are exposed (Table 1):

**Table 1.** The 10 generic chain of custody phases as established by Law No. 13.964 of 2019 and their descriptions.

Phase	Description
I—Evidence identification	Act of distinguishing an element as of potential interest for the production of expert evidence.
II—Scene security	Act of preventing the state of affairs from changing, and must secure and preserve the primary, secondary and related environment^3^ to the traces and crime scene.
III—Evidence documentation	Detailed description of the trace as found at the crime scene or in the corpus delicti analysis, and its position in the examination area, which can be illustrated by photographs, footage or sketches, its description being essential in the expert report produced by the expert responsible for the service.
IV—Evidence collection	Act of collecting the trace that will be subjected to expert analysis, respecting its characteristics and nature.
V—Evidence packaging	Procedure whereby each collected trace is individually packaged, according to its physical, chemical and biological characteristics, for further analysis, with annotation of the date, time and name of the person who carried out the collection and packaging.
VI—Evidence transportation	Act of transferring the trace from one place to another, using the appropriate conditions (packaging, vehicles, temperature, among others), in order to guarantee the maintenance of its original characteristics, as well as the control of its possession.
VII—Evidence receipt	Formal act of transfer of possession of the trace, which must be documented with at least information regarding the case number and related police unit, place of origin, name of who transported the trace, tracking code, nature of the examination, type of trace, protocol, signature and identification of who received it.
VIII—Evidence processing	Expert examination itself, manipulation of the trace according to the methods appropriate to its biological, physical and chemical characteristics, in order to obtain the desired result, which must be formalized in a report produced by an expert.
IX—Evidence storage	Procedure referring to the storage, in appropriate conditions, of the material to be processed, kept for inspection, disposed or transported, linked to the corresponding report number.
X—Evidence disposal	Procedure regarding the release of the trace, respecting current legislation and, when relevant, upon judicial authorization.

### Evidence identification

The chain of custody begins with police or expert procedures in which the existence of a trace is detected. The first public security agent must be able to recognise that a crime was committed and identify the elements of potential interest.

The ability to identify evidence of forensic anthropological interest is one of the crucial points for the investigation to follow the procedures in accordance with expert jurisprudence. It is therefore an essential condition for the other steps in the processing of a crime scene to be fully successful.

It is recommended that an expert who works or has knowledge in ​​forensic anthropology is present at the crime scene, so that the anthropological evidence is identified correctly, and the scene security is adequately modified to ensure the integrity and legitimacy of the traces *in situ*. In other words, a forensic anthropology expert can assure that any physical evidence not detected by unknowledgeable persons is identified and protected for posterior collection.

Investigating a crime scene with proper identification of forensic anthropological evidence is crucial for the protection, recovery, and collection of such materials of interest. Evidence identification, as a first step, allows for the accurate collection and sorting of evidence that paves the way for an overall successful investigation. Ideally, forensic anthropologists should always take part in the crime scene investigation, using their experience to analyse the scene and recognise its associated materials as possible matters of investigative interest. The value of a forensic anthropologist at the scene can be exemplified when there is a need for excavation, identification of human or non-human remains, recognition of bone elements and fragments, interpretation of the scene and its context, and assurance that complete and thorough recovery of evidence is accomplished.

The main risk during the evidence identification phase is the biological risks regarding the state of decomposition of the cadaver. In addition, there is the risk associated with the lack of knowledge when identifying materials, such as bone elements or fragments, and the inability to recognise a site as being of secondary deposition of human remains.

The complex nature of sites, such as possible fires, landslides, mixed graves, and disasters, is the main vulnerability of the process. The search, location, and identification of traces can be hampered by the presence of other materials, by the fragile state of the traces of interest, and by the extent of the area to be surveyed.

### Scene security

The public security agent who recognises an element as being of potential interest to experts is directly responsible for its preservation. The chain of custody processes then follows with the preservation of the crime scene.

As soon as a public security institution becomes aware that a criminal offense has occurred, the police authority must go to the location and ensure that the scene is properly preserved until the arrival of the official criminal experts. They must also determine, if applicable, that an examination of the *corpus delicti* and any other analysis is carried out. For the purpose of examining the location where the offense was committed, the authority will immediately ensure that the situation does not change until the arrival of the official experts who will subsequently illustrate their reports with photographs, drawings, or clarifying schemes. The experts will record in the report the changes in the situation and will discuss the consequences of these changes in the dynamics of the facts.

Entry of unauthorized persons into secured places is prohibited, as is the removal of any traces of crime scenes before release by the responsible official expert. The alteration of a crime scene is typified as procedural fraud^4^.

Complementing the evidence identification phase, scene security has the important function of preventing access to materials of anthropological interest by people who may intend to subtract or alter them. Therefore, the scene must contain physical means to limit access, such as cones or striped tapes, and the perimeter must be monitored by public security professionals.

One of the most important aspects of protecting the crime scene is to preserve it with minimal contamination and disturbance of the traces present. The first responder(s) must promptly remain alert to any people, vehicles, events, potential traces, and environmental conditions. The safety and physical well-being of officers and other individuals in and around the crime scene are the priorities of the initial responding officer(s).

With the arrival of the crime scene official criminal expert and sometimes the official expert in the areas of forensic medicine or legal odontology, it is possible that the original scene protection method will be changed after a wide sweep. It may aim to cover a more extensive area or other areas that were recognised as related to the event.

The main risk and vulnerability of non-existent or inadequate scene security is the removal of disassociated bone elements or other evidence associated with the event (such as projectiles and biological evidence) or the contamination of existing traces.

### Evidence documentation

The human remains should always be photographed in the position in which they were found, as well as all external injuries and traces left at the scene of the crime. To represent the lesions found on the human remains, the official experts, when possible and considering their knowledge and limitations, should add photographic evidence, diagrams, or drawings to the examination report.

As part of the procedures carried out when human remains are found, steps involving the survey of the environmental conditions of the location should be described. The vegetation, humidity, and type of soil in the region should be observed, with attention given to searching for traces that indicate the occurrence of any anthropogenic changes in the examined environment. There must be a detailed search for bones and their fragments, in addition to teeth that may have detached from the body after skeletonisation. The topography of the searched region should be considered. The surface layer of the soil can be sifted to search for bones and teeth and other traces that may possibly be associated with elements of interest found in the bones, such as projectiles expelled by firearms. Furthermore, it is essential that photographs are taken of the scene and any traces or evidence found. The positions of any traces should be associated with the location in accordance with the methodology that the official expert deems most pertinent.

**Figure 2.**
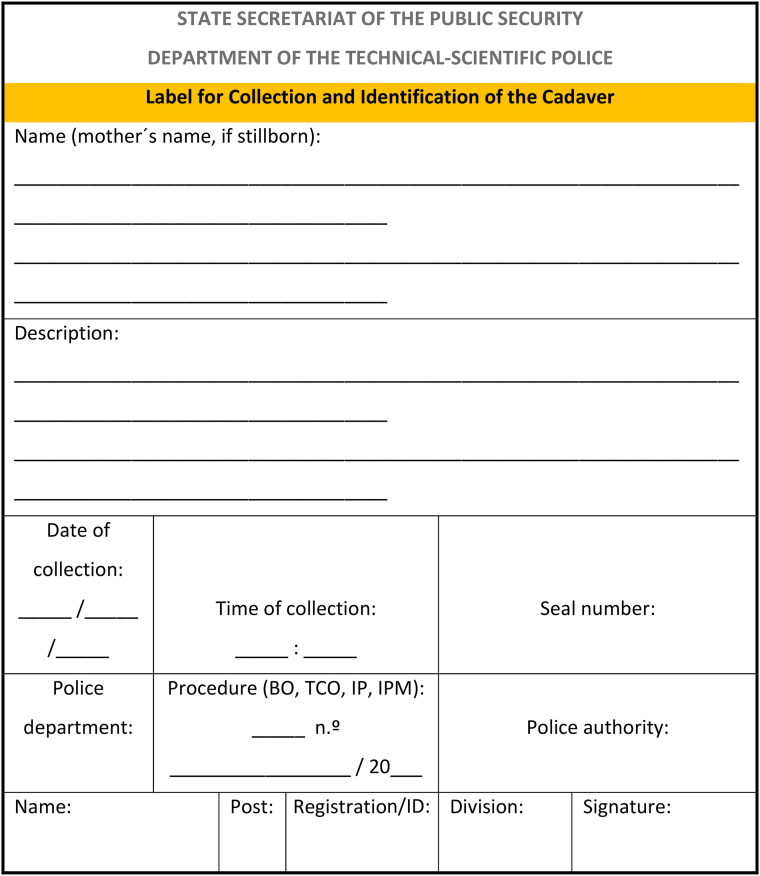
Example of an identification and collection tag/label.

After completing the peri-necroscopic^5^ examination, the official expert at the scene should ideally attach a waterproof identification and collection tag/label (Figure 2) to a location/body segment that, at their discretion, allows for better adherence, observation, and preservation of the tag. This label must minimally contain information related to the police procedure number, the date and time of collection, the qualification of the responsible expert, the expert unit to which they belong, and a brief description of the evidence. Once attached, the label must be photographed with at least two images, one allowing legible reading of the information and the other covering the immediate surrounding context.

Any trace associated with the human remains that may come off from its body surface or body cavities must be photographed *in situ*, collected, and packaged individually in an envelope that must be sealed following the general procedures, keeping the related specificities according to the nature of the evidence. Associated traces that the official expert believes do not run the risk of detachment from the cadaver or human remains should also be marked as an observation in the TTF.

Incorrect or inadequate documentation may generate inconsistent information or lack of information necessary for the report and its context, especially if it is going to be collected and processed, after which such information cannot be supplied. This inadequacy could possibly be brought up in court to render the evidence inadmissible of evidence or lessen its value and impact.

There is great vulnerability when teams process the scene without the basic knowledge necessary to identify traces as of forensic anthropological interest, compromising their documentation and posterior collection.

### Evidence collection

The collection of traces should preferably be carried out by an official expert, who will provide the necessary referral to the Custody Centre even when additional complementary analyses are required. All traces collected during the investigation must be treated as described in the legal and criminal procedures, with the central body of official criminal expertise being responsible for detailing the manner of compliance.

The cadaver/human remains may still have clothing or other materials, such as wrappings, associated materials, or covers, that can be partially or completely removed from the scene at the discretion of the official expert. They will assess the indispensability of such action for the examination of the body and the limitations related to the context, such as improper exposure of the body. Items will be removed after being packed and deposited in a mortuary bag or paper bag, then will be duly registered in the TTF. If the official expert believes traces could possibly be lost, they will be packed in a proper envelope or bag to be sealed. The proper TTF will then be completed, where the reason for its removal from the scene will be recorded and the type of trace collected will be specified, such as a blood stain or semen sample.

The step described above is subject to careful documentation. Two concerns of forensic anthropological analysis are the registration of evidence origin and its context. Provenance refers to the exact location of an item in three-dimensional space, reflecting the latitude, longitude, and vertical positioning. Context is the exact time and space of an object considering its association and relationship to other items. Undoubtedly, this information is lost the moment objects are collected, as the investigation and collection process impact a scene from the moment they begin. Official experts have only one chance to extract traces completely and correctly, occasionally referred to as “controlled deconstruction”.

The effective collection of the trace is dependent on its documentation *in situ*, emphasising that all these data must be consulted later during the writing of the report. Although there is no strict order for the process of collecting traces, some types of materials must take precedence. For example, traces that are transient, fragile, or can be easily lost have priority. When dealing with disassociated human remains or fragments of bodies, each element must be treated as unique. In situations of graves with multiple bodies, experts without proper forensic anthropological knowledge must avoid reassociating disassociated bone elements. Emphasis must be given to the documentation phase, so that information can be retrieved later for more specialised analysis.

The risks and vulnerabilities during the collection of traces of forensic anthropological interest at the scene arise from the very nature of this evidence. They are often fragile and associated with other potential traces. Compromising the integrity of the traces during collection can also result in contamination of other traces and expose the expert team to biological risks.

### Evidence packaging

The receptacle^6^ used for packaging of the trace will be determined by the nature of the material being collected. All containers must be sealed with individual numbering to guarantee the inviolability and suitability of the evidence during transport. It must individualise the evidence, preserve its characteristics, prevent contamination and leakage, and have an adequate degree of resistance and space for recording information about its content. The receptacle can only be opened by the official expert who is going to perform the analysis or, by an authorised person designated to perform such action. After each time the seal is broken, the name and registration of the person responsible, date, place, purpose, and information regarding the new seal must be included in the TTF. The broken seal must be packed inside the new container. Along with labelling, two records are needed to maintain the chain of custody: an inventory of the recovered material and a record of people who came into contact with the evidence.

The cadaver (or human remains in different stages of decomposition) will be placed in an impermeable mortuary bag with adequate resistance to loading the body. The bag should preferably have at least four handles for safe collective loading of its contents, ensuring its integrity and greater ergonomics for those responsible for carrying it, have two zippers or a zipper and associated device at the end that makes it possible to seal the bag to guarantee its inviolability and suitability. This zipper, together with the seal, will have a copy of the label/tag that was attached to the body.

It is recommended that skeletonised remains, disassociated elements, bones, fragile materials, and clothing be packed in paper bags to prevent any moisture that can result in fungi or mold formation, which would hinder the further processing and analysis of these elements. Each element/trace collected must be immediately placed in an appropriate primary container and then into a secondary container, which must be completely sealed with tape.

The official expert responsible for the scene processing must at least supervise the disposition of the cadaver/human remains in the mortuary bag, personally close the bag, fasten the seal to the zippers or zipper/closure device, and place the corresponding label. Once the mortuary bag is sealed, the official expert must take at least two photographs of the installed seal and the label attached to it, which must clearly show the printing on the label, the specific numbering of the seal, and the immediate surrounding context. After placing the mortuary bag in the tray of the transport vehicle and verifying the documents, the person responsible for transport must fill out and sign the item related to the transport information contained in the TTF. The official expert must then proceed to photograph the completed FAV.

### Evidence transportation

All receptacles must be sealed with individual numbering to guarantee the inviolability and suitability of the trace during transport.

The removal of human remains from the crime scene will only take place after the completion of the criminal investigation activities and the subsequent authorisation of the person responsible for the judicial police work. The crime scene expert must supervise the collection of all materials and ensure that the human remains have been deposited in the transport compartment tray to preserve its integrity. The vehicles of the IML fleet, funeral homes, or other companies contracted for this purpose must have an adapted cargo transport compartment with appropriate individualised trays and adequate refrigeration to ensure the integrity of the human remains.

Professionals responsible for transport should check the information on the FAV and its consistency with the material to be transported. They must also observe the specific conditions for transport, which will be described in the TTF, as well as instructions for filling out specific data in the TTF related to transport. Any non-conformity must be reported to the official expert at the scene and, if the non-conformity(ies) persists, they must be recorded, specified, and reported to the official expert at the scene in the respective TTF.

The IML, whenever possible, should promptly respond to requests for the collection of cadavers/human remains. When it is not possible to quickly respond, the responsible unit must immediately inform the police authority of this circumstance, stating the reasons for impediment.

The vehicle used for transport, after collecting the cadaver, human remains, and/or associated evidence of a crime scene, must go immediately and directly to the destination IML without unnecessary stops or detours. When this does not occur due to force majeure (e.g. weather or traffic), the reason(s) or complications must be reported in the TTF. Likewise, any intercurrences that may change the conditions of transport or prevent the guidelines given by the responsible official expert from being followed will be reported and justified by the carrier at the FAV.

When transporting bone elements, fragile, fractured, or charred bones, the possibility of using additional cushioning systems during packaging and transport should be considered to preserve the integrity of this evidence. The guidelines will be included in the FAV of the evidence and will be passed on to the person responsible for the transport.

### Evidence receipt

The person responsible for receiving the evidence must check the TTF, paying special attention to any recommendation regarding transport/storage, such as special care given to fragile bones or loose teeth. After verification, the person responsible should register the receipt with the TTF, note the time at which it occurred and point out any inconsistencies between the information and the mortuary bag and/or containers with other traces. Such non-conformities must be registered in the TTF by the professional and immediately communicated to the police authority.

Ideally, the cadaver, human remains, and/or associated evidence should be received properly packaged in sealed bags and accompanied by the respective TTFs. The absence of the recommended recipients or bags, while weakening the chain of custody, is not a sufficient reason for its total breakage or for the inadmissibility in processing the evidence being received. Unless such packaging poses a risk to the integrity of the traces or especially to personnel or infrastructure, it is recommended that traces be accepted and received in containers other than the standardised ones, especially if minimally adequate and with justification. Care should be taken to register any such non-conformity in the necessary documents.

For the entry of human remains into the IML through its Custody Centre, it must be formally registered in an “electronic medical record” (or a physical notebook) with the IML internal tracking number, which can be used as a tracking code. If a new number is given, it is essential that the document refers to the original case number and the number of the TTF that accompanied the traces from the original scene to the IML.

The registration of the cadaver in a computerised system (or a physical notebook) must include the following information:

- Location TTF number—unique numbering;
- Case number and related judicial police unit;
- Place of origin;
- Person responsible for transporting the human remains;
- Tracking code/protocol number—internal to IML;
- Nature of the exam requested;
- Identification and signature of the person responsible for receiving the body.

Upon receiving the request order for the examination, the IML Custody Unit must verify the agreement with the information contained on the plastic seal and label of the mortuary bag, in the “electronic medical record” (or proper book), and in the TTF of the human remains.

In situations where the IML does not have an infrastructure or human resources capable of performing the specialised examination, it must receive the cadaver/human remains and arrange transport to another IML, recording this in the TTF and informing the requesting authority.

Upon receipt by the Custody Centre (morgue), the mortuary bag can only have its seal broken and the human remains exposed by the official expert(s) of the forensic medicine or Legal Odontology area designated for the examination.

### Evidence processing

The sealed recipient can only be opened by the official expert who is going to conduct the requested analysis and, with good reason, by an authorised person. After each time the seal is broken, the name and registration of the person responsible, date, place, purpose, and information regarding the new seal used must be included in the TTF. The broken seal must be packed inside the new container.

The peri-necroscopic examination (also considered to be evidence processing) will be carried out by the official expert at the scene and is part of the processes related to the crime scene itself. The limitations related to the context, their professional ethics, previous experience, and professional background should be considered.

When evidence processing takes place at the IML, other chain of custody phases also occur concomitantly, such as the identification and documentation phases. The official expert makes the selection and determines if any evidence received is of interest to Forensic Anthropology. In addition to the types of evidence previously described as having forensic anthropological interest, cases of cadavers/human remains where it is not possible to perform a conventional autopsy must be referred for anthropological examination. This can occur at either the same institute or a separate location that has a specialised Forensic Anthropology service.

The official expert responsible for the forensic anthropological analyses should start the necroscopic examination by verifying the bag/seal and note any nonconformities in the respective FAV. The official forensic expert in the medical/odontology area will also be responsible for collecting, storing, and forwarding the associated evidence (traces of interest in pathology, ballistics, sexology, and toxicology, for example). Care must be taken to allow for the discovery of additional traces, such as entomological or ballistic evidence, during processing. Any additional traces must be treated in accordance with the procedures applicable for their nature and any necessary referrals to other laboratories must be made by filling out the appropriate documentation.

If identification of the cadaver/human remains is possible, information regarding missing persons should be requested to compare with *postmortem* data. Preferably, this standard documentation should be received directly by the Forensic Anthropology service at the IML, where interviews with family members are also carried out. This documentation must, whenever possible, be digitised and stored in the institution’s system and/or in the Custody Unit (digital, if any). It must also contain the human remains data, TTF number, and internal IML tracking code.

If the human remains require reconstruction for better examination during analysis, the process and materials used must be properly documented. Any methods of reconstruction or materials used must be reversible with non-destructive ways to aid documentation. That means that the methods for assembling fragmented bones should be reversible by using an adequate solvent, as in the case of inadequate gluing, or of a necessary modification when an additional bone fragment is found.

Adequate laboratory lighting is crucial for traces to be analysed effectively and efficiently. Lighting also assists in the documentation of evidence, providing that the images produced are trustworthy to the analysed physical trace.

The parallel documentation phase that occurs with the evidence processing within the IML (intra-institutional) consists of photographic records of the trauma, pathology, and elements used for the biological profile evaluation, individualising characteristics, or any alterations present in the human remains. Photography is the simplest and most effective way to perform this step. More than any hand-written representation, photography can provide important details for elucidating the individual’s identity and cause and manner of death. It is difficult to determine the ideal number of photos that should be taken. However, all records should be made together with the internal record number of the remains. In general, photos should preferably be orthogonal, in small, medium, and large scale, where the character or feature of interest is clearly depicted.

Another effective form of documentation is an imaging exam. Radiographs make it possible to identify bone fractures and locate firearm projectiles or other objects foreign to the human body. Computed tomography (CT) and magnetic resonance imaging (MRI), which are still not widely used in forensic sciences, make it possible to perform re-examinations through image reconstruction without the need for exhumation of the body in some situations. All images or videos generated must include the human remains data, TTF number, and internal IML tracking code. This documentation should, whenever possible, be digitised and stored in the institution’s system and/or in the Custody Unit.

The description and documentation of the analysed material is of paramount importance because some processing methods are destructive, such as the removal of soft tissue. All procedures, equipment, techniques, and methodologies relevant to the processing of anthropological evidence and present in the anthropological report must be described and include any appropriate scientific references.

For bodies at different stages of decomposition and not completely skeletonised, it is recommended that caution be used during the maceration process. Because of differences in the laboratory infrastructure, it is understood that total body maceration and cleaning often become infeasible. Therefore, it is recommended that only the essential elements for specific analyses and estimates of the biological profile are macerated and cleaned. These elements can include the pelvis (pubis), skull, long bone (particularly of the leg, femur, or tibia), and any element that is of interest for analysis of trauma or pathology.

After the procedures have been finished or any technical issues have been identified, the official expert who performed the analysis/breaking of the seal must supervise the repackaging of the human remains in a mortuary bag (or archival skeletal remains box) and reseal it. The cadaver/human remains will only return to the Custody Centre in a duly sealed mortuary bag (or archival skeletal remains box) with a new number registered in the FAV by the official criminal expert.

### Evidence storage

All Criminalistics Institutes must have a Custody Centre for the custody and control of traces, and its management must be directly linked to the central body of official criminal expertise.

Every Custody Centre must have protocol services with a place for conference, reception, and return of materials and documents, enable the selection, classification, and distribution of materials, and be a safe space with environmental conditions that do not interfere with the characteristics of the evidence. At the Custody Centre, the entry and exit of traces must be filed and all information pertinent to the investigation, including the case number, must be recorded. All persons who have access to the stored trace must be identified and the date and time of access must be noted. When processing the stored trace, all actions must be registered, including the identification of the person responsible for processing, as well as the destination, date, and time of the action.

After the specialised analysis has been conducted, the material must be returned to the Custody Centre and must remain there. If the Custody Centre does not have space or conditions to store certain materials, the police or judicial authority shall determine the conditions for storing said material in a different location upon request of the director of the central institution of official criminal expertise.

In laboratory settings, experts will keep enough material for the possibility of a new analysis request. If there is potential evidential value, the skeleton, bone elements, or associated evidence must be safely kept at the Ossuary. A plastic burial box, archival skeletal remains box, or similar container should preferably be used for storage, provided they have perforations in each end of the lid that allow them to be properly sealed. It is recommended that two seals with unique numbers are provided to preserve the inviolability of the box. The box containing the evidence must be accompanied by the respective TTF.

In cases of violent death, clothes and other belongings with evidential value that have been removed from the human remains at the time of the necropsy will be packaged, registered in their own TTFs, and stored at the Custody Centre of general traces until they are processed. After processing/analysis, the responsible official expert will return the post-processed/remaining material to the Custody Centre, which will be communicated to the responsible Police Authority. Clothes and belongings removed from the cadaver at the time of necropsy that have no recognised forensic interest will be packaged, identified, and sent to the Custody Centre for general traces. They will remain at that location and be available for withdrawal by the family/representative(s) for a period of 15 days, after which they will be properly discarded.

After the forensic analysis of the traces, their respective TTFs will be stored at the Custody Centre in an area where employees can exclusively access them. An efficient cataloguing and searching system will be maintained. If there is interest from the expert institute and/or the Public Administration, for example, if they are awaiting complementary examinations or *antemortem* data, the skeleton/bone elements may be kept in custody at the Ossuary for any time that is justified by its current or potential evidential value. If the Custody Centre of the IML does not have space or conditions to store the bones or other traces, the police or judicial authority must be informed to determine the conditions for storing them in a different location, upon request of the IML Director.

### Evidence disposal

In this proposed model, the traces must be transported to the Custody Centre for final storage until their disposal. There is an exception in some special situations, such as specific evidence like cadavers and bones, where the disposal management will be coordinated by the Custody Centre and carried out directly by the Ossuary.

The person responsible for releasing the cadaver/human remains must check the documentation that accompanies the body/bone elements and consult in the reporting system and with the previous responsible official expert, if there are any pending issues. The release of the cadaver/human remains must be included in the respective item/sub-item in the TTF. The document must also contain the status of the cadaver/human remains (identified, visually recognised, or unidentified)^7^ and signature of the person removing the body such as a relative or the funeral home.

Disposal of human remains refers to the procedures for releasing, donating, cremating, or burying them. The procedures should follow the laws and regulations of the country, as well as state decrees that vary throughout Brazil. Identified cadavers/remains can be delivered to either the first-degree family members or their legal representatives or to other relatives who legally request the cadavers/remains, pending judicial authorisation. Unclaimed cadavers/remains can be buried or donated to other institutions (academia or research) after the specific number of wait days legally established by the State. Human remains can only be cremated if they have been positively identified and the deceased individual has specified their cremation intention in legal documents in cases of natural death or with a judicial police authorisation in cases of violent death. In cases of unidentified bodies, the importance of controlling the burial site and subsequent storage in the cemetery’s ossuary is highlighted, following the same recommendations as the Ossuary in the IML. This allows for easy location of them if a future identification becomes necessary. Furthermore, the State allows for the donation of unidentified cadavers/remains. However, the authors do not recommend the donation of unidentified human remains for ethical and social reasons.

Skeletal material is often kept by the forensic anthropologist in secure storage facilities until the responsible investigative authority has made the final determination for their disposal. Bones that show evidence of specific *peri-mortem* trauma, or that may assist in establishing an identity in a case where future positive identification is likely or that a previous identification can be contested, may be retained after the rest of the remains are discarded. This can only be done, however, with the express permission of the Chief Medical Examiner and with the knowledge of the investigators. The chain of custody must then reflect which bones were returned and which were retained.

## Final considerations

Evidence of forensic anthropological interest can involve human remains or bodies in conditions of a complex nature, including bodies that can be burned, fragmented and in advanced stages of decomposition. This evidence can be located both on the surface or buried in graves and is generally very sensitive. Forensic anthropological evidence can be associated with crime scenes involving violent deaths, accidents, fires, disasters, and situations of a political or humanitarian context as in human rights violations.

Therefore, it is important to detail aspects relevant to the implementation and maintenance of the chain of custody of forensic anthropological evidence. The actions of the professionals involved in the movement or manipulation of the evidence in question must also be standardised. It is noteworthy that the multidisciplinary work involving the interactions between different agencies and actors of Public Security and other institutions and their specific responsibilities need to be coordinated and well defined to avoid interference. This can prevent the investigation from being compromised.

This article presents the first recommendations for the evidence chain of custody in forensic anthropology in Brazil, which will help the development of the field. The importance of following the recommendations proposed here is highlighted, based on minimum standardised practices, to avoid compromising the evidence. Furthermore, this document can generally guide institutes and agencies with varied technical capacities and resources with publications of protocols, decrees, ordinances, circulars, and other internal legal norms that are well defined and publicised. This complements and helps apply these and other recommended practices and procedures.

It is suggested that all phases of the chain of custody process described here are strictly followed, and that it is understood that phases can sometimes occur simultaneously, such as evidence identification, documentation, and processing. If it is impossible to comply with any of the recommendations, the noncompliance must be registered and documented as part of the chain of custody.

The authors understand that the chain of custody goes beyond the insurance of documental registration and traceability. The weakening of the chain of custody, even when there is no break in the traceability of the evidence, impacts the analysis of the sameness and appropriateness of the evidence. This precariousness and instability, in turn, impact the conditions of the evidence during its processing and, concomitantly, may alter its natural state. Hence, to maintain the robustness and admissibility of the evidence, we must ensure that adequate methodologies and techniques are being applied by proper specialised professionals following the considerations also established by *Daubert*.

Furthermore, it is recommended that the actions of the personnel involved are ideally standardised to preserve the inviolability and suitability of traces related to forensic anthropology, safeguarding their evidential value. Therefore, adapting the procedures correctly is essential to strengthen the expert evidence, which must be reputable and lawful, and to mitigate possible legal questions regarding the evidence and methodological and technical procedures applied throughout the investigation.

## Notes

1 Pronouns added by the authors. Original sentence only brings “he”.

2 In this model, the Custody Units are extensions of the Custody Centre, administratively linked to the respective Forensic Institutes to which they belong, and normatively linked to the Custody Centre. This is in turn directly linked to the central official forensic agency of criminal nature.

3 Primary environment: location that generated the first activation of public security forces, which may be the place where the criminal event took place, the result, or even an act after the criminal conduct. This can include places of concealment of a cadaver, but is somehow related to the criminal event. Secondary environment: the intermediate area between the place where the event took place and the large external environment that may contain traces related to the fact under investigation. Between the direct and indirect environments there is a geographic continuity. Related environment: any place with no direct geographic connection to the crime scene (previously classified as direct/indirect) and which contains some trace related to the fact.

4 It is one of the crimes against the administration of justice. It consists of artificially innovating, pending civil or administrative proceedings, the state of place, thing, or person to mislead the judge or expert. The penalty provided is imprisonment for 3 months to 2 years and a fine.

5 Examination of a cadaver or human remains *in situ* at the scene.

6 Or container.

7 “Identified” refers to the status of an individual reached through methods that are scientifically sound (such as DNA or fingerprint analysis); “Visually recognized” is a status of an individual reached by a witness account that may suggest an identity, but is not sufficient for positive identification, being merely empirical and generally validated by the police authority via a document called a recognition report (auto de reconhecimento, in Portuguese); “Unidentified” is a status of an individual with no positive identity reached. Based on: Guide, D.V.I., 2014. INTERPOL Disaster Victim Identification Guide. *Mars*.
